# PROTOCOL: The effectiveness of community, financial, and technology platforms for delivering nutrition‐specific interventions in low‐ and middle‐income countries: A systematic review

**DOI:** 10.1002/cl2.1037

**Published:** 2019-09-08

**Authors:** Amynah Janmohamed, Nazia Sohani, Zohra S Lassi, Zulfiqar A Bhutta

**Affiliations:** ^1^ Centre for Global Child Health The Hospital for Sick Children Toronto Canada; ^2^ Robinson Research Institute University of Adelaide Adelaide Australia

## 1 BACKGROUND

About half of global under‐5 child mortality, or about 3 million deaths, are linked to poor nutrition (UN Inter‐agency Group for Child Mortality, 2017; UNICEF, 2018). The effects of compromised nutrition at an early age are evident throughout the life course, with physical and cognitive impairments affecting health, learning, and economic potential (Martins, Toledo Florêncio, & Grillo, 2011). Good nutrition is also important beyond the childhood years, with adolescent girls being especially vulnerable to undernutrition because of their higher nutritional requirements, particularly those who might become pregnant. Therefore, a focus on adolescent girls’ nutrition is important to ensure adequate prepregnancy nutrition for maternal, fetal, and infant health. Evidence suggests under‐5 child mortality can be reduced by 15% with 90% coverage of 10 evidence‐based nutrition interventions (Bhutta, Das, & Rizvi, 2013). However, despite evidence of efficacy generated from controlled settings, the potential impacts of what are considered “proven” nutrition interventions are often not realized in real‐world environments due to ineffective delivery channels for achieving high and equitable coverage. A review by Ramakrishnan et al. (2014) noted that while prenatal protein‐energy and iron folic acid supplementation have been shown to reduce low birth weight by 20–30% in trial settings, variable implementation has led to uncertain effectiveness. Menon et al. (2014) also acknowledge evidence supporting effective delivery platforms for nutrition‐specific interventions remains limited. Of particular concern are gaps in how to successfully reach adolescents with evidence‐based nutrition interventions in low‐ and middle‐income countries (LMIC) (Bhutta, Lassi, & Bergeron, 2017; Salam, Hooda and Das, 2016). Our review considers delivery platforms that can improve coverage of nutrition‐specific interventions at all stages of the life course from preconception to pregnancy, infancy, childhood, and adolescence. The review is part of a series of concurrent reviews to produce up‐to‐date evidence on preventive and curative nutrition interventions across the lifecycle.

### 1.1 Description of the condition

The review will consider platforms for interventions to address a variety of nutrition‐specific conditions. We will examine the effects of using community, financial, and technology platforms for delivering evidence‐based nutrition‐specific interventions to improve nutrition behaviors and outcomes for women, children, and adolescents in LMICs. For the purpose of our review, a “platform” is defined as a modality through which a service is made available to target beneficiaries.

### 1.2 Description of the intervention

#### 1.2.1 Description of platforms

In this review, we have chosen to limit our focus to community, financial, and technology‐based platforms for providing direct nutrition interventions to populations in LMICs. These platforms are widely used globally and were reviewed previously (Bhutta et al., 2013). While acknowledging the existence of other health delivery platforms, we have limited our review to those that integrate a direct nutrition component for feasibility reasons.

##### 1.2.1.1 Community platforms

Community delivery platforms have shown potential for increasing coverage of evidence‐based nutrition interventions and improving equity of service delivery (Bhutta et al., 2013). In LMICs, these platforms include community health workers (CHWs), peer groups (women and mothers), and community outreach events (e.g., Child Health Days [CHD]) that provide health and nutrition services at the community level. In many countries, extending the reach of the health system has involved training CHWs to deliver essential low‐cost health and nutrition interventions such as counseling on prenatal nutrition and appropriate breastfeeding and complementary feeding practices, micronutrient supplementation, and child growth monitoring, with evidence indicating properly trained CHWs can improve key maternal, infant, and child nutrition practices (Bhutta, Lassi, Pariyo, & Huicho, 2009; Perry, Zulliger, & Rogers, 2014; Shakir, 2010). Community platforms are also important channels for reaching adolescent girls who are less likely to seek preventive care at health care facilities. CHD are a widely used community platform in Sub‐Saharan Africa (SSA) and involve semiannual provision of an integrated package of child and family health and nutrition interventions such as micronutrient supplementation, immunization, deworming, and insecticide‐treated bednets (UNICEF, 2017). CHDs have been particularly successful for increasing coverage of vitamin A supplementation for children <5 years in SSA (Oliphant, Mason, & Doherty, 2010). We will review the evidence for CHWs, CHDs, and similar events, as well as peer group models (and other community platforms identified in our search) as a means to increase coverage and impact of nutrition interventions targeted to women, children, and adolescents in LMICs.

##### 1.2.1.2 Financial incentive platforms

Nutrition‐sensitive programs can improve the coverage and effectiveness of nutrition‐specific interventions (Ruel, Alderman, Maternal, & Child Nutrition Study Group, 2013). Financial incentive platforms are increasingly being used in LMICs as part of poverty‐reduction/social protection programs to reduce economic barriers to achieving better health and nutrition outcomes through enabling higher quality diets, increased access to health services, and improved living environments (de Groot, Palermo, Handa, Ragno, & Peterman, 2015). These mainly consist of cash payments or vouchers targeted to poor households, and commonly to mothers of young children. While evidence suggests the potential positive impact of conditional cash transfers, where cash is provided to beneficiaries upon compliance with health and/or nutrition‐promoting services (e.g., child growth monitoring, nutrition education sessions), for improving coverage of child health interventions such as breastfeeding practices, the quality of available evidence is low and evidence gaps remain (Bassani et al., 2013; Bastagli, Hagen‐Zanker, & Harman, 2016; Lagarde, Haines, & Palmer, 2009). We will review the evidence on the nutritional effects of financial incentive platforms (involving a nutrition‐related conditionality) that are targeted to women and children in LMICs.

##### 1.2.1.3 Technology platforms

The review will include technology platforms, given their increasing relevance for nutrition interventions in LMICs. Though there is broad clinical application for technology to improve health in these settings through telemedicine and other telehealth services for diagnosis and treatment, we focus on key technology platforms for nutrition promotion, including mass and social media and mobile health. The use of mobile phone technologies, such as SMS messaging, has shown to be effective for improving health‐related behaviors through facilitating greater connectivity between providers and communities in remote areas (Barnett, Yosellina, & Sulistyo, 2016; Källander, Tibenderana, & Akpogheneta, 2013). Mass media involves dissemination of health information through traditional radio spots, print material, and television broadcasts. Social media utilizes internet‐based applications such as websites, blogs, and so forth, to promote healthy practices and behaviors and has great potential for reaching adolescents. Given the growing penetration of mobile phones in low‐resource settings and increased global connectivity via the Internet, these platforms are increasingly being leveraged for nutrition programming in LMICs (Tamrat & Kachnowski, 2012). We will review the evidence for these platforms as means to deliver interventions targeted to women, children, and adolescents in LMICs.

### 1.3 How the intervention might work

**Figure 1:**
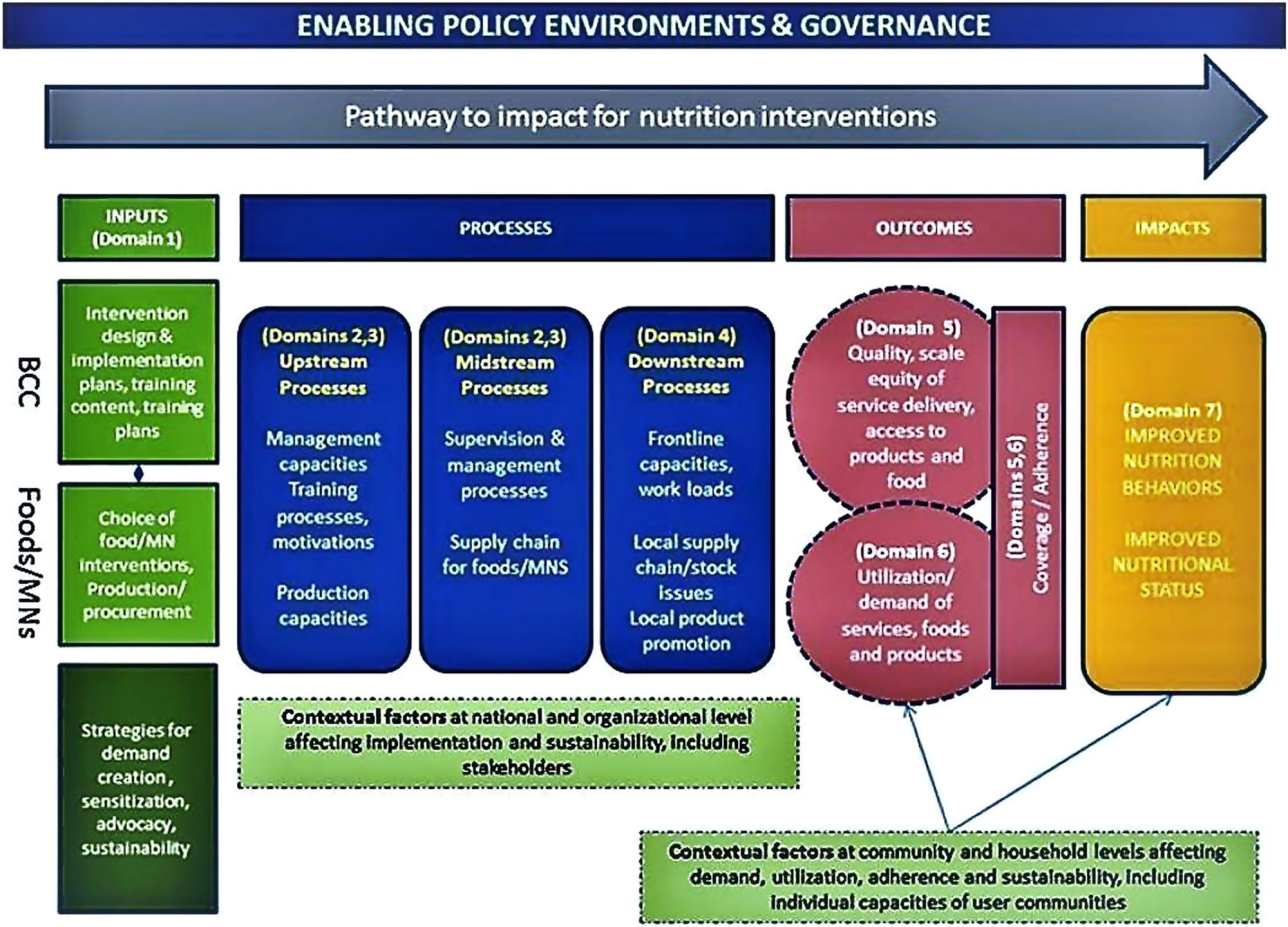
Nutrition implementation framework [Color figure can be viewed at wileyonlinelibrary.com]

Health and nutrition gains are contingent on how well interventions are targeted, implemented, and utilized in a particular context. To guide our review, we use Menon et al. (2014) Nutrition Implementation Framework (Figure 1) as our theory of change model. The framework considers core implementation domains affecting quality of service delivery, coverage, utilization, and impact with a view to scaling‐up prioritized nutrition interventions. Though a range of nutrition‐specific interventions can potentially be delivered through our included platforms, common interventions include counseling and education for women and mothers on good maternal nutrition and optimal infant and young child feeding practices through community outreach efforts such as home visits and peer group sessions, as well as media events and other community mobilization activities.

### 1.4 Why it is important to do this review

Improving nutrition in LMICs requires investments in “proven” interventions, as well as knowledge of effective mechanisms for delivering high‐impact interventions to those most in need as, without good coverage, even the most efficacious interventions will not achieve impact at scale. Though the merits of using specific platforms (e.g., CHWs, cash transfers) for health and nutrition are well‐described in the literature and have been shown through efficacy studies, the effectiveness of nutrition interventions is likely to vary depending on the delivery platform. Our review aims to review and synthesize evidence on key delivery platforms that are effective for improving coverage, utilization, and or impact (nutrition benefit gained) from nutrition‐specific interventions targeted to women, children, and adolescents in LMICs. In combination, coverage, utilization, and impact are considered “effective” coverage (Ng, Fullman, & Dieleman, 2014). Where possible, we will assess *effective* coverage, but will also examine components of effective coverage separately depending on data available.

A key focus of the review will build on prior evidence suggesting CHWs are important agents to improving uptake of child nutrition interventions in hard‐to reach populations. The 2013 *Lancet* nutrition series (Bhutta et al., 2013) concluded community delivery strategies that reach poor at‐risk segments of the population have potential to increase population‐level coverage of nutrition interventions through demand creation and household service delivery. Further, in a review of 82 studies, Lewin et al. (2010) showed positive effects of lay health workers for promoting the initiation of breastfeeding (risk ratio [RR], 1.36; 95% confidence interval [CI]: 1.14–1.61) and exclusive breastfeeding (RR, 2.78; 95% CI: 1.74–4·44), when compared with the standard of care.

Our review will provide up‐to‐date evidence to help inform policy and programming for delivery of nutrition‐specific interventions to promote health and well‐being through improved nutrition behaviors and practices in LMICs, while also highlighting gaps in the existing evidence surrounding the effectiveness of community, financial, and technology platforms requiring further study.

## 2 OBJECTIVES

The objectives of the review are as follows:

1. To assess the coverage of nutrition‐specific interventions delivered using community, financial, and technology platforms
2. To assess the utilization of nutrition‐specific interventions delivered using community, financial, and technology platforms
3. To assess the nutritional impact of nutrition‐specific interventions delivered using community, financial, and technology platforms

## 3 METHODS

### 3.1 Criteria for considering studies for this review

#### 3.1.1 Types of studies

We will include primary studies, including large‐scale program evaluations, that use a community, financial, or technology platform to deliver a nutrition‐specific intervention using one of the following study designs:

1. Randomized controlled trials (RCTs) where participants were randomly assigned, individually or in clusters, to intervention and comparison groups (includes cluster and stepped‐wedge RCTs).
2. Quasiexperimental studies in which nonrandom assignment to intervention and comparison groups was based on other known allocation rules, including a threshold on a continuous variable (regression discontinuity designs) or exogenous geographical variation in the treatment allocation (natural experiments)
3. Controlled before‐after studies in which allocation to intervention and control groups was not made by study investigators, but outcomes were measured in both intervention and control groups pre‐ and post‐intervention and appropriate methods were used to control for selection bias and confounding such as statistical matching (e.g., propensity score matching, covariate matching) or regression adjustment (e.g., difference‐in‐differences, instrumental variables). Pre‐post studies without a control group will not be included.
4. Interrupted time series studies in which outcomes were measured in the intervention group at a minimum of three time points before and after the intervention.

#### 3.1.2 Types of participants

The target populations for this review are pregnant women, mothers of children <5 years, children <5 years, children 5–9 years, and female adolescents 10–19 years living in a LMIC as defined by the World Bank (see below). Studies including both eligible and noneligible participants will only be included if we can disaggregate relevant data.

#### 3.1.3 Types of interventions

We will include experimental studies and program evaluations that report coverage, utilization, and/or impact of nutrition‐specific interventions. Interventions to be examined in our review are based on evidence‐informed recommendations to reduce poverty and knowledge barriers. Many of these interventions are behavioral, such as education and support to mothers to promote early and exclusive breastfeeding and appropriate complementary feeding practices, and can be delivered through multiple platforms. For example, interventions that include an education component could be delivered within the context of community‐based nutrition promotion programs or through large mass media campaigns. However, each platform‐intervention combination will be synthesized separately. Interventions will be compared against the standard of care in respective settings and we will exclude studies that do not have a control group. If a study includes multiple intervention arms, we will only include those meeting our eligibility criteria. Interventions to be included for each platform are presented in Table 2.

#### 3.1.4 Types of outcome measures

The primary outcomes are coverage, utilization, and impact of nutrition interventions. Eligible outcome measures by platform are summarized in Table 2. All outcomes will be measured separately by target group and platform. For example, a breastfeeding promotion intervention may be provided to both adolescent mothers and women of reproductive age (WRA) using different platforms. Further, in the context of a breastfeeding promotion intervention, we are interested in studies that report the percentage of mothers reached with breastfeeding counseling, the uptake of improved breastfeeding practices, and if available, the effect of the improved practice on the child's nutritional status (e.g., infant growth as assessed by weight gain, height gain, *Z* scores for height‐for‐age (HAZ), weight‐for‐height (WHZ), weight‐for‐age (WAZ), stunting, wasting, underweight). Definitions for primary outcomes are presented below.

##### 3.1.4.1 Primary outcomes

Coverage: the proportion of a population that is eligible to benefit from an intervention that actually receives it.

Outcome example 1: proportion of targeted mothers of children <5 years receiving at least one monthly home visit from a CHW.

Outcome example 2: proportion of targeted women receiving at least 90 iron folic acid tablets during pregnancy.

Utilization: the proportion of the eligible population that receives and adopts an intervention (i.e., uptake, intended change in behavior observed)

Outcome example 1: proportion of targeted infants breastfed within one hour of birth.

Outcome example 2: proportion of targeted children 6–23 months of age receiving minimum meal frequency.

Impact: the health benefit/gain experienced by the target population as a result of the intervention; here we will focus on anthropometric and micronutrient status outcomes for all groups.

Outcome example 1: proportion of targeted infants wasted (WHZ < −2*SD*) at 12 months of age.

Outcome example 2: Average hemoglobin measurement in adolescent girls pre‐ and postintervention.

Table 2 includes the primary outcome indicators to be measured by platform, intervention, and target population. There will be no restrictions based on duration of exposure or timing of outcome measurement. For studies that have varying time points for outcome measurement, we will include and report all time points, using the time point that is most similar across studies for data synthesis.

We do not expect adverse outcomes given the nature of the nutrition‐specific interventions delivered through community, financial, and technology platforms (e.g., education).

##### 3.1.4.2 Secondary outcomes

We will not examine secondary outcomes in the review.

##### 3.1.4.3 Duration of follow up

There will be no restrictions regarding duration of follow‐up.

##### 3.1.4.4 Types of settings

Included studies will have been conducted in a LMIC, as defined by the World Bank (2018), at the time of publication. Low‐income economies are defined as those with a gross national income (GNI) per capita of USD 1,005 or less in 2016 and lower middle‐income economies are countries with a GNI per capita between USD 1,006 and 3,955 in 2016. Local settings will comprise urban, rural, or mixed environments. Depending on the study context, an intervention may be delivered in a micro‐level environment (e.g., community village education) or a macro‐level environment (e.g., provincial cash transfer program).

### 3.2 Search methods for identification of studies

#### 3.2.1 Electronic searches

Our search strategy is guided by our PICO model Table 1 and will not be restricted by outcome. For indexed databases, the search will be conducted using medical subject headings and free text key words. The search strategy specific to each database is provided in Appendix 1. We will also review reference lists of included papers and relevant reviews for eligible studies. Studies published during 1997 to June 2018 will be included and studies published in languages other than English will be excluded due to resource limitations. Clinicaltrials.gov and WHO's ICRTP will be searched for ongoing trials.

**Table 1:** PICO model

Population	WRA, pregnant women, mothers of children <5 years, children <5 years, children 5–9 years, adolescents 10–19 years living in a low‐ and middle‐income countries
Intervention	Nutrition‐specific intervention delivered using a community, financial, or technology‐based platform
Comparison	Author‐defined
Outcome	Primary:
	1. Coverage 2. Utilization 3. Impact

**Table 2:** Eligible interventions, populations and primary outcome measures, summarized by platform

Platform	Mode of delivery	Intervention	Population	Coverage measure (%)	Utilization measure (%)	Impact measure
Community	Community health worker	Breastfeeding education (home visits, community sessions)	Pregnant women, mothers of newborns, mothers of children 6–23 months	Proportion of women receiving breastfeeding education during prenatal, postpartum, and 0–12 month period	– Proportion of infants breastfed within an hour of birth – Proportion of infants <6 months exclusively breastfed – Proportion of infants receiving breast milk at 12 months	Anthropometric measure (stunting, wasting, underweight, mean HAZ, WHZ, WAZ); hemoglobin, anemia, iron, vitamin A status
	Community health worker	Complementary feeding education (home visits, community sessions)	Mothers of children 6–23 months	Proportion of women receiving complementary feeding education	– Proportion of children given solid or semisolid foods at least the minimum number of times according to age and breastfeeding status – Proportion of children with minimum dietary diversity – Proportion of children consuming specific foods (animal‐source, iron/vitamin A‐rich foods)	Anthropometric measure (stunting, wasting, underweight, mean HAZ, WHZ, WAZ); hemoglobin, anemia, iron, vitamin A status
	Community health worker	Provision of maternal iron folic acid supplementation	Pregnant women, mothers of newborns (for postpartum IFA supplementation)	Proportion of women receiving IFA during prenatal and postpartum period	Proportion of women consuming IFA supplements	Hemoglobin, anemia, iron status
	Community health worker	Provision of child micronutrient supplementation (e.g., vitamin A, multiple micronutrient sachet)	Children <5 years	Proportion of children <5 receiving supplements	Proportion of children consuming micronutrient supplements	Hemoglobin, anemia, iron status, vitamin A status
	Peer support groups	Promotion of nutrition messages through discussions and demonstrations	WRA, pregnant women, mothers of children <5	Proportion of women attending peer group sessions	Proportion of women complying with specific behavior	Reported nutritional status indicators
	Community outreach campaigns (e.g., Child Health Days)	Nutrition education, provision of micronutrient supplementation	WRA, pregnant women, mothers of children <5, children <5, adolescents	Proportion of target population reached	Proportion of target population complying with specific behavior	Reported nutritional status indicators
Financial incentives	Conditional cash transfers, conditional vouchers (conditioned on a nutrition‐related behavior)	WRA, pregnant women, mothers of children <5 in eligible households	Proportion of eligible recipients meeting set condition	Proportion of targeted individuals complying with specific behavior	Reported nutritional status indicators	
Technology	mHealth	Nutrition education, reminder messaging (e.g., for taking prenatal IFA tablets)	WRA, pregnant women, mothers of children <5, adolescents	Proportion of target population reached	Proportion of targeted individuals complying with specific behavior	Reported nutritional status indicators
	Mass media	Nutrition education	WRA, pregnant women, mothers of children <5, adolescents	Proportion of target population reached	Proportion of targeted individuals complying with specific behavior	Reported nutritional status indicators
	Social media	Nutrition education	WRA, pregnant women, mothers of children <5, adolescents	Proportion of target population reached	Proportion of targeted individuals complying with specific behavior	Reported nutritional status indicators

Abbreviations: HAZ, height‐for‐age *Z* score; IFA, iron and folic acid; WRA, women of reproductive age; WAZ, weight‐for‐age *Z* score; WHZ, weight‐for‐height *Z* score.

We will search the following electronic reference databases/libraries based on their relevance to the topic under review:

- ClinicalTrials.gov
- Embase
- MEDLINE
- Scopus
- Web of Science
- WHO e‐Library of Evidence for Nutrition Actions (eLENA)
- WHO Global Database on the Implementation of Nutrition Action (GINA)
- WHO International Clinical Trials Registry Platform
- WHO library database (WHOLIS)

#### 3.2.2 Searching other resources

Our search will include studies outside the peer‐reviewed literature (e.g., nonindexed program evaluations). To retrieve such documents, we will use key words to search the following websites: Global Alliance for Improved Nutrition, International Food Policy Research Institute, International Initiative for Impact Evaluation (3ie), Nutrition International, UNICEF, USAID and affiliates (e.g., FANTA, SPRING), World Bank, and the World Food Programme. We will also search the ProQuest database for dissertations.

### 3.3 Data collection and analysis

#### 3.3.1 Selection of studies

Two review authors will independently screen titles and abstracts using prespecified inclusion and exclusion criteria. Any article selected by at least one reviewer will be included for further screening. All full texts will be screened in duplicate by review authors using the same criteria, with reasons for exclusion recorded. Discrepancies will be resolved by a third reviewer. Title/abstract and full text screening will be conducted using Covidence.

#### 3.3.2 Data extraction and management

Data extraction will be conducted in duplicate by two review authors using a common data extraction form following pre‐specified instructions and decision rules, including standardized conventions for data coding and recording with preset form entries.The data extraction form will be piloted and the following study information will be extracted:

- General study information: title, authors, publication year, type of study design, funding source.
- Study setting: country, World Bank region, World Bank income category (low‐ or lower‐middle income) at time of publication, city/town, urban/urban slum/rural/mixed setting.
- Study population: age range, median, mean + *SD* age, characteristics (e.g., pregnant), sex, ethnicity, sample size recruited, sample size analysed (for cluster trials: number of clusters and number of people per cluster), significant baseline imbalances
- Delivery platform (e.g., CHW, CHD, cash transfer, etc.).
- Characteristics of each intervention: number of intervention groups, type of intervention, unit of randomization (if applicable), setting (e.g., facility, home), timing, frequency, duration, duration of follow‐up, attrition rate (and reasons if provided).
- Quantitative outcomes (coverage, utilization, impact): outcome measures in intervention and comparison group (unit and *SD*); time points measured; effect measure (95% CI, *SE*, *p* value); subgroup outcome measures (if applicable)
- Qualitative outcomes (targeting, implementation fidelity): entered as descriptive text
- Study quality assessment results

If study information is unclear or cannot be obtained from the paper, we will contact the authors for further details. Missing information will be noted as not available.

#### 3.3.3 Assessment of risk of bias in included studies

The risk of bias for included studies will be assessed in duplicate, with inconsistencies resolved by a third review author. For RCTs, the Cochrane risk of bias tool (Higgins et al., 2016) will be used. For RCTs, we will assess risk of bias according to the following domains and rate each as either “low risk,” “high risk,” or “unclear risk” with justifications.

- Selection bias (random sequence generation, allocation concealment)
- Performance bias
- Detection bias
- Attrition bias
- Reporting bias
- Other risks of bias

For non‐RCTs controlled before‐after studies, and interrupted time series, we will use the EPOC tool (Cochrane Effective Practice and Organisation of Care [EPOC], 2017) to assess risk of bias according to the following domains and rate each as either “low risk,” “high risk,” or “unclear risk” with justifications.

- Baseline characteristics similar
- Baseline outcome measurements similar
- Knowledge of the allocated interventions adequately prevented
- Protection against contamination
- Selective outcome reporting
- Other risks of bias

#### 3.3.4 Measures of treatment effect

We will analyse dichotomous and continuous outcomes separately. For dichotomous outcomes, effect measures will be reported as relative risks or odds ratios with 95% CIs. We will present continuous outcome data as either a mean difference (MD), if outcomes have been measured on the same scale, or a standardized mean difference, if outcomes have been measured on different scales, with 95% CIs. Both change scores and final measurement values will be eligible and can be pooled for meta‐analyses with MD.

#### 3.3.5 Unit of analysis issues

If an outcome is reported using different metrics, we will perform unit conversions (i.e., g/dl to g/L for hemoglobin or mm to cm for height) in order to pool data using methods described in the *Cochrane Handbook* (Higgins & Green, 2011). Where possible for continuous measures, similar effect sizes will be transformed to indicate the same direction (positive estimate). For cluster RCTs, we will ensure clustering has been appropriately accounted for in the analysis of the primary study, such that study precision is not over or under‐estimated in our analysis. If necessary, we will adjust effect estimates of cluster‐randomized trials by applying the design effect using the mean cluster size (*M*) and the intracluster correlation coefficient (ICC) [design effect = 1 + (*M* − 1) ICC]. The design effect will be used to adjust the study data such that a trial is reduced to its effective sample size. We will not make any adjustments if authors have appropriately adjusted for clustering. We will conduct a sensitivity analysis whereby Hedges’ *g* bias‐corrected estimates are used to correct for upward bias associated with small sample sizes (<20).

#### 3.3.6 Dealing with missing data

If authors account for missing data (e.g., multiple imputations), we will use the adjusted values. If necessary, we will contact study authors to request missing data, clarifications for missing data, or to request data in a more usable format for the review. Reasons for missing data will be documented.

#### 3.3.7 Assessment of heterogeneity

Statistical heterogeneity will be assessed using τ^2^, *I* ^2^ and significance of the χ^2^ test; we will also assess heterogeneity visually using forest plots. Any observed outliers, also assessed through visual inspection of the forest plots, will be discussed within the findings. Based on prior theory and clinical knowledge, we expect clinical and methodological heterogeneity in effect sizes. Therefore, we will attempt to explain any observed statistical heterogeneity using subgroup analyses (see below).

#### 3.3.8 Assessment of reporting biases

If the number of studies is sufficient (>10), funnel plots will be used to visually assess publication bias. This type of bias is unlikely if data form a symmetric inverted funnel shape around the mean effect estimate. In addition, we will perform Egger's test to determine funnel plot asymmetry.

#### 3.3.9 Data synthesis

We will prepare a matrix of all studies grouped by platform, intervention, population, outcome, and study design to examine data suitable for meta‐analyses. On the basis of the prior literature review, outcomes of interest include early initiation of breastfeeding (within 1 hr), exclusive breastfeeding (to 6 months), diet‐related indicators of minimum dietary diversity, minimum meal frequency, and minimum acceptable diet, iron folic acid and vitamin A supplementation. Impact measures include stunting, wasting, underweight, and continuous mean HAZ, WHZ, and WAZ measures. These will be analysed separately. Additional outcomes may be synthesized where data permit. Where this occurs, we will note the posthoc selection of outcomes.

Depending on data availability, outcomes that differ along a continuum of length of follow‐up will be grouped according to similar follow‐up time points. On the basis of the previous literature, we do not expect follow‐up times to be >24 months so we will include the latest follow‐up time for each study. We will list the primary outcome for each comparison with the estimate of relative effect and the number of participants for studies contributing data for those outcomes. If studies include data that cannot be pooled, we will retain the study as eligible but restrict it from further analysis.

We will conduct separate meta‐analyses for different study designs (RCTs vs. nonrandomized studies) and for subcategories of platforms, interventions and outcomes. We will not combine continuous and dichotomous effect size data and will conduct separate meta‐analyses for these measures. We will conduct random‐effects meta‐analyses, given the diversity of study contexts, participants, interventions, and so forth. Effect sizes and standard errors will be meta‐analyzed using the inverse variance method in RevMan 5.3 (RevMan, 2014). Where meta‐analysis is not appropriate due to substantial heterogeneity, findings will be summarized in narrative/table form to describe patterns in direction of effect and size of effect reported, noting factors that might explain differences in effects across included studies. For interpretation of results, we will consider effect estimates that have associated *p* < .05 as statistically significant. We will also report nonsignificant findings. Statistical analysis will be performed using RevMan 5.3.

We will construct a “Summary of findings” table for all primary outcomes that includes quality of evidence. The quality of evidence will be rated according to GRADE criteria (Guyatt, Oxman, & Akl, 2011): within‐study risk of bias (methodological quality), directness of evidence, heterogeneity, precision of effect estimates, and risk of publication bias. We will rate the quality of the body of evidence for each outcome as “high,” “moderate,” “low,” or “very low.” Evidence can be upgraded for outcomes with a large magnitude of effect, presence of a dose‐response relationship, and or accounting for the effect of plausible residual confounding. Evidence will be downgraded if there is risk of bias in individual studies, indirectness of evidence, unexplained heterogeneity, imprecision of results, or a high probability of publication bias.

#### 3.3.10 Dependency

Potential sources of dependency will be taken into consideration. If there are two or more papers describing the same study, they will be combined and coded as a single study. For trials that include multiple eligible intervention arms, we will select one pair (intervention and control) that meets our inclusion criteria. Inclusion of other relevant pairs will be considered in a sensitivity analysis. If studies include more than one target population (each with an intervention and control arm), then data will be disaggregated into corresponding subgroups and may be included in the same forest plot.

#### 3.3.11 Subgroup analysis and investigation of heterogeneity

Statistical heterogeneity will be assessed using τ^2^, *I* ^2^ and significance of the χ^2^ test. We will also assess heterogeneity visually using forest plots. If necessary, meta‐regression will be considered to examine the influence of moderator variables on effect size. Depending on data availability (three studies per characteristic), we will consider the following subgroup analyses:

- Area (urban vs. rural)
- Age (0–5 months, 6–11 months, 12–23 months, 24–59 months, 5–9 years, 10–19 years, >19 years)
- Sex
- Baseline nutritional status (e.g., anemic vs. nonanemic; underweight vs. not underweight)
- Socio‐economic status
- Ethnicity
- Frequency/duration of intervention (e.g., <3, 3–6, 6–12 months)

#### 3.3.12 Treatment of qualitative research

This review will not include qualitative research studies.

## DECLARATIONS OF INTEREST

The authors are not aware of any conflicts of interest arising from financial or researcher interests.

## SOURCES OF SUPPORT

Funding for this review came from a grant from the Bill & Melinda Gates Foundation to the Centre for Global Child Health at The Hospital for Sick Children (Grant No. OPP1137750).

## APPENDIX 1 Search strategy

**Table:**

Database [Platform]
*MEDLINE*
Ovid MEDLINE(R) Epub ahead of print, In‐Process and Other Non‐Indexed Citations, Ovid MEDLINE(R) Daily and Ovid MEDLINE(R)
*Embase Classic + Embase*
*Web of Science*
Science Citation Index Expanded (SCI‐EXPANDED)
Social Sciences Citation Index (SSCI)
Conference Proceedings Citation Index‐Science (CPCI‐S)
Conference Proceedings Citation Index‐Social Science & Humanities (CPCI‐SSH)
Emerging Sources Citation Index (ESCI)
*EBM Reviews‐Cochrane Central Register of Controlled Trials*

### MEDLINE

Ovid MEDLINE(R) Epub ahead of print, In‐Process and Other Nonindexed Citations, Ovid MEDLINE(R) Daily and Ovid MEDLINE(R)

Search Strategy:

**Table:**

#	Searches
1	(pediatric* or paediatric* or child* or newborn* or congenital* or infan* or baby or babies or neonat* or “pre‐term” or preterm* or “premature birth*” or NICU or preschool* or “pre‐school*” or kindergarten* or kindergarden* or “elementary school*” or “nursery school*” or (“day care*” not adult*) or schoolchild* or toddler* or boy or boys or girl* or “middle school*” or pubescen* or juvenile* or teen* or youth* or “high school*” or adolesc* or “pre‐pubesc*” or prepubesc*).tw,kf. or (child* or adolesc* or pediat* or paediat*).jn.
2	Prenatal care/ or Perinatal care/ or obstetrics/ or breast feeding/
3	women/ or pregnant women/
4	(obstetric* or gynecolog* or gynaecolog* or perinatal or prenatal or “pre natal” or antenatal or “ante natal” or postnatal or “post natal” or “maternal health” or gestation or pregnancies or pregnant or pregnancy or childbearing or gravidity or mother* or breastfeed* or “breast feeding” or woman or women).tw,kf.
5	or/1–4
6	Developing Countries.sh,kf.
7	(Africa or Asia or Caribbean or “West Indies” or “South America” or “Latin America” or “Central America”).tw,kf,hw,cp.
8	(Afghanistan or Albania or Algeria or Angola or Antigua or Barbuda or Argentina or Armenia or Armenian or Aruba or Azerbaijan or Bahrain or Bangladesh or Barbados or Benin or Byelarus or Byelorussian or Belarus or Belorussian or Belorussia or Belize or Bhutan or Bolivia or Bosnia or Herzegovina or Hercegovina or Botswana or Brasil or Brazil or Bulgaria or “Burkina Faso” or “Burkina Fasso” or “Upper Volta” or Burundi or Urundi or Cambodia or “Khmer Republic” or Kampuchea or Cameroon or Cameroons or Cameron or Camerons or “Cape Verde” or “Central African Republic” or Chad or Chile or China or Colombia or Comoros or “Comoro Islands” or Comores or Mayotte or Congo or Zaire or “Costa Rica” or “Cote d'Ivoire” or “Ivory Coast” or Croatia or Cuba or Cyprus or Czechoslovakia or “Czech Republic” or Slovakia or “Slovak Republic” or Djibouti or “French Somaliland” or Dominica or “Dominican Republic” or “East Timor” or “East Timur” or “Timor Leste” or Ecuador or Egypt or “United Arab Republic” or “El Salvador” or Eritrea or Estonia or Ethiopia or Fiji or Gabon or “Gabonese Republic” or Gambia or Gaza or “Georgia Republic” or “Georgian Republic” or Ghana or “Gold Coast” or Greece or Grenada or Guatemala or Guinea or Guam or Guiana or Guyana or Haiti or Honduras or Hungary or India or Maldives or Indonesia or Iran or Iraq or “Isle of Man” or Jamaica or Jordan or Kazakhstan or Kazakh or Kenya or Kiribati or Korea or Kosovo or Kyrgyzstan or Kirghizia or “Kyrgyz Republic” or Kirghiz or Kirgizstan or “Lao PDR” or Laos or Latvia or Lebanon or Lesotho or Basutoland or Liberia or Libya or Lithuania or Macedonia or Madagascar or “Malagasy Republic” or Malaysia or Malaya or Malay or Sabah or Sarawak or Malawi or Nyasaland or Mali or Malta or “Marshall Islands” or Mauritania or Mauritius or “Agalega Islands” or Mexico or Micronesia or “Middle East” or Moldova or Moldovia or Moldovian or Mongolia or Montenegro or Morocco or Ifni or Mozambique or Myanmar or Myanma or Burma or Namibia or Nepal or “Netherlands Antilles” or “New Caledonia” or Nicaragua or Niger or Nigeria or “Northern Mariana Islands” or Oman or Muscat or Pakistan or Palau or Palestine or Panama or Paraguay or Peru or Philippines or Philipines or Phillipines or Phillippines or Poland or Portugal or Puerto Rico or Romania or Rumania or Roumania or Russia or Russian or Rwanda or Ruanda or “Saint Kitts” or “St Kitts” or Nevis or “Saint Lucia” or “St Lucia” or “Saint Vincent” or “St Vincent” or Grenadines or Samoa or “Samoan Islands” or “Navigator Island” or “Navigator Islands” or “Sao Tome” or “Saudi Arabia” or Senegal or Serbia or Montenegro or Seychelles or “Sierra Leone” or Slovenia or “Sri Lanka” or Ceylon or “Solomon Islands” or Somalia or “South Africa” or Sudan or Suriname or Surinam or Swaziland or Syria or Tajikistan or Tadzhikistan or Tadjikistan or Tadzhik or Tanzania or Thailand or Togo or “Togolese Republic” or Tonga or Trinidad or Tobago or Tunisia or Turkey or Turkmenistan or Turkmen or Uganda or Ukraine or Uruguay or USSR or “Soviet Union” or “Union of Soviet Socialist Republics” or Uzbekistan or Uzbek or Vanuatu or “New Hebrides” or Venezuela or Vietnam or “Viet Nam” or “West Bank” or Yemen or Yugoslavia or Zambia or Zimbabwe or Rhodesia).hw,kf,ti,ab,cp.
9	((developing or “less* developed” or “under developed” or underdeveloped or “middle income” or “low* income” or underserved or “under served” or deprived or poor*) adj1 (countr* or nation? or population? or world)).tw.
10	((developing or “less* developed” or “under developed” or underdeveloped or “middle income” or “low* income”) adj1 (economy or economies)).tw.
11	(low* adj1 (gdp or gnp or “gross domestic” or “gross national”)).tw.
12	(low adj3 middle adj3 countr*).tw.
13	(lmic or lmics or “third world” or “lami countr*”).tw.
14	“transitional countr*”.tw.
15	or/6–14
16	5 and 15
17	exp mass media/ or exp social media/ or blogging/ or social networking/ or radio/
18	(Internet or “cyber space” or cyberspace or “world wide web” or “information and communication technologies” or “communication technolog*” or ICTs or “communication application*” or “communication apps”).tw,kf.
19	((virtual or online) adj2 (communit* or network* or forum? or support*)).tw,kf.
20	(“mass media” or “social media” or “social medium?” or “social network*” or sns or multimedia).tw,kf.
21	(phone adj2 (app? or application?)).tw,kf.
22	(“push technolog*” or “web 2.0” or “web 2.0 s” or radio or television* or TV or TVs or twitter or facebook or WeChat or weibo or YouTube or whatsapp or blogging or blog? or “hash tag*” or hashtag* or microblog* or “chat room” or “Internet chat” or “online chat” or email or wiki*).tw,kf.
23	(sms or “short messag*” or text* or “mobile outreach” or “mobile out reach” or mms or smartphone* or telephone* or cellphone*).tw,kf.
24	((text or short or instant or multimedia or “multi‐media”) adj1 messag*).tw,kf.
25	((car or cell or cellular or mobile or smart) adj1 (phone or phones or telephone*)).tw,kf.
26	(mobile adj2 communication).tw,kf.
27	(mhealth or “m‐health” or ehealth or “e‐health” or “mobile medicine” or “mobile health”).tw,kf.
28	or/17–27
29	16 and 28
30	((cash or money or monetary or electronic) adj1 (transfer* or assistant* or voucher* or allowance?)).tw,kf.
31	(“mobile money” or “mobile pay” or “e‐transfer” or “etranfer” or “financial assistance” or “financial support” or “food stamp?” or “food voucher?” or “food assistance” or “cash credit”).tw,kf.
32	food supply/ or (“food insecurity*” or “food security” or “food suppl*” or “social protection”).tw,kf.
33	or/30–32
34	16 and 33
35	(“community health” adj1 (service? or centre? or center? or promotion or “out reach” or outreach)).tw,kf.
36	((“health care” or healthcare or health) adj1 (worker? or personnel or volunteer* or provider* or network or campaign* or alliance)).tw,kf.
37	((community or “community based”) adj1 (program* or network* or mobili?er or “out reach” or outreach or aide? or involvement or participation)).tw,kf.
38	(“volunt* worker?” or “peer group?” or “wom#n group?” or “wom#n's group?” or “child health day” or “integrated management of childhood illness” or IMCI).tw,kf.
39	or/35–38
40	16 and 39
41	29 or 34 or 40
42	(“macro‐nutrient” or “macro nutrient” or macronutrient or malnutrition or malnourishment or intracortical* or undernutrition or undernourishment or underfeeding or malabsorption or nourish*).tw,kf.
43	(diet? or dietary or nutrition* or supplement* or food? or feed*).tw,kf.
44	or/42–43
45	41 and 44
46	limit 45 to yr = “1997 ‐Current”
47	limit 46 to English language

*Embase Classic + Embase*

Search Strategy:

**Table:**

#	Searches	
1	(pediatric* or paediatric* or child* or newborn* or congenital* or infan* or baby or babies or neonat* or “pre‐term” or preterm* or “premature birth*” or NICU or preschool* or “pre‐school*” or kindergarten* or kindergarden* or “elementary school*” or “nursery school*” or (“day care*” not adult*) or schoolchild* or toddler* or boy or boys or girl* or “middle school*” or pubescen* or juvenile* or teen* or youth* or “high school*” or adolesc* or “pre‐pubesc*” or prepubesc*).tw,kw. or (child* or adolesc* or pediat* or paediat*).jn.
2	exp prenatal care/ or exp perinatal care/ or exp obstetrics/ or exp breast feeding/ or female/ or pregnant women/
3	(obstetric* or gynecolog* or gynaecolog* or perinatal or prenatal or “pre natal” or antenatal or “ante natal” or postnatal or “post natal” or “maternal health” or gestation or pregnancies or pregnant or pregnancy or childbearing or gravidity or mother* or breastfeed* or “breast feeding” or woman or women).tw,kw.
4	or/1–3
5	Developing Countries.mp.
6	(Africa or Asia or Caribbean or “West Indies” or “South America” or “Latin America” or “Central America”).tw,kw,hw,cp.
7	(Afghanistan or Albania or Algeria or Angola or Antigua or Barbuda or Argentina or Armenia or Armenian or Aruba or Azerbaijan or Bahrain or Bangladesh or Barbados or Benin or Byelarus or Byelorussian or Belarus or Belorussian or Belorussia or Belize or Bhutan or Bolivia or Bosnia or Herzegovina or Hercegovina or Botswana or Brasil or Brazil or Bulgaria or “Burkina Faso” or “Burkina Fasso” or “Upper Volta” or Burundi or Urundi or Cambodia or “Khmer Republic” or Kampuchea or Cameroon or Cameroons or Cameron or Camerons or “Cape Verde” or “Central African Republic” or Chad or Chile or China or Colombia or Comoros or “Comoro Islands” or Comores or Mayotte or Congo or Zaire or “Costa Rica” or “Cote d'Ivoire” or “Ivory Coast” or Croatia or Cuba or Cyprus or Czechoslovakia or “Czech Republic” or Slovakia or “Slovak Republic” or Djibouti or “French Somaliland” or Dominica or “Dominican Republic” or “East Timor” or “East Timur” or “Timor Leste” or Ecuador or Egypt or “United Arab Republic” or “El Salvador” or Eritrea or Estonia or Ethiopia or Fiji or Gabon or “Gabonese Republic” or Gambia or Gaza or “Georgia Republic” or “Georgian Republic” or Ghana or “Gold Coast” or Greece or Grenada or Guatemala or Guinea or Guam or Guiana or Guyana or Haiti or Honduras or Hungary or India or Maldives or Indonesia or Iran or Iraq or “Isle of Man” or Jamaica or Jordan or Kazakhstan or Kazakh or Kenya or Kiribati or Korea or Kosovo or Kyrgyzstan or Kirghizia or “Kyrgyz Republic” or Kirghiz or Kirgizstan or “Lao PDR” or Laos or Latvia or Lebanon or Lesotho or Basutoland or Liberia or Libya or Lithuania or Macedonia or Madagascar or “Malagasy Republic” or Malaysia or Malaya or Malay or Sabah or Sarawak or Malawi or Nyasaland or Mali or Malta or “Marshall Islands” or Mauritania or Mauritius or “Agalega Islands” or Mexico or Micronesia or “Middle East” or Moldova or Moldovia or Moldovian or Mongolia or Montenegro or Morocco or Ifni or Mozambique or Myanmar or Myanma or Burma or Namibia or Nepal or “Netherlands Antilles” or “New Caledonia” or Nicaragua or Niger or Nigeria or “Northern Mariana Islands” or Oman or Muscat or Pakistan or Palau or Palestine or Panama or Paraguay or Peru or Philippines or Philipines or Phillipines or Phillippines or Poland or Portugal or Puerto Rico or Romania or Rumania or Roumania or Russia or Russian or Rwanda or Ruanda or “Saint Kitts” or “St Kitts” or Nevis or “Saint Lucia” or “St Lucia” or “Saint Vincent” or “St Vincent” or Grenadines or Samoa or “Samoan Islands” or “Navigator Island” or “Navigator Islands” or “Sao Tome” or “Saudi Arabia” or Senegal or Serbia or Montenegro or Seychelles or “Sierra Leone” or Slovenia or “Sri Lanka” or Ceylon or “Solomon Islands” or Somalia or “South Africa” or Sudan or Suriname or Surinam or Swaziland or Syria or Tajikistan or Tadzhikistan or Tadjikistan or Tadzhik or Tanzania or Thailand or Togo or “Togolese Republic” or Tonga or Trinidad or Tobago or Tunisia or Turkey or Turkmenistan or Turkmen or Uganda or Ukraine or Uruguay or USSR or “Soviet Union” or “Union of Soviet Socialist Republics” or Uzbekistan or Uzbek or Vanuatu or “New Hebrides” or Venezuela or Vietnam or “Viet Nam” or “West Bank” or Yemen or Yugoslavia or Zambia or Zimbabwe or Rhodesia).tw,kw,hw,cp.
8	((developing or “less* developed” or “under developed” or underdeveloped or “middle income” or “low* income” or underserved or “under served” or deprived or poor*) adj1 (countr* or nation? or population? or world)).tw.
9	((developing or “less* developed” or “under developed” or underdeveloped or “middle income” or “low* income”) adj1 (economy or economies)).tw.
10	(low* adj1 (gdp or gnp or “gross domestic” or “gross national”)).tw.
11	(low adj3 middle adj3 countr*).tw.
12	(lmic or lmics or “third world” or “lami countr*”).tw.
13	“transitional countr*”.tw.
14	or/6–13
15	4 and 14
16	mass communication/ or blogging/ or e‐mail/ or hotline/ or Internet/ or mass medium/ or exp mobile phone/ or radio/ or social media/ or telephone/ or television/ or text messaging/
17	(Internet or “cyber space” or cyberspace or “world wide web” or “information and communication technologies” or “communication technolog*” or ICTs or “communication application*” or “communication apps”).tw,kw.
18	((virtual or online) adj2 (communit* or network* or forum? or support*)).tw,kw.
19	(“mass media” or “social media” or “social medium?” or “social network*” or sns or multimedia).tw,kw.
20	(phone adj2 (app? or application?)).tw,kw.
21	(“push technolog*” or “web 2.0” or “web 2.0s” or radio or television* or TV or TVs or twitter or facebook or WeChat or weibo or YouTube or whatsapp or blogging or blog? or “hash tag*” or hashtag* or microblog* or “chat room” or “Internet chat” or “online chat” or email or wiki*).tw,kw.
22	(sms or “short messag*” or text* or “mobile outreach” or “mobile out reach” or mms or smartphone* or telephone* or cellphone*).tw,kw.
23	((text or short or instant or multimedia or “multi‐media”) adj1 messag*).tw,kw.
24	((car or cell or cellular or mobile) adj1 (phone or phones or telephone*)).tw,kw.
25	(mobile adj2 communication).tw, kw.
26	(mhealth or “m‐health” or ehealth or “e‐health” or “mobile medicine” or “mobile health”).tw,kw.
27	or/16–26
28	15 and 27
29	((cash or money or monetary or electronic) adj1 (transfer* or assistant* or voucher* or allowance?)).tw,kw.
30	(“mobile money” or “mobile pay” or “e‐transfer” or “etranfer” or “financial assistance” or “financial support” or “food stamp?” or “food voucher?” or “food assistance” or “cash credit”).tw,kw.
31	(“food insecurity*” or “food security” or “food suppl*” or “social protection”).tw,kw.
32	or/29–31
33	15 and 32
34	(“community health” adj1 (service? or centre? or center? or promotion or “out reach” or outreach)).tw,kw.
35	((“health care” or healthcare or health) adj1 (worker? or personnel or volunteer* or provider* or network or campaign* or alliance)).tw,kw.
36	((community or “community based”) adj1 (program* or network* or mobili?er or “out reach” or outreach or aide? or involvement or participation)).tw,kw.
37	(“volunt* worker?” or “peer group?” or “wom#n group?” or “wom#n's group?” or “child health day” or “integrated management of childhood illness” or IMCI).tw,kw.
38	or/34–37
39	15 and 38
40	28 or 33 or 39
41	(“macro‐nutrient” or “macro nutrient” or macronutrient or malnutrition or malnourishment or intracortical* or undernutrition or undernourishment or underfeeding or malabsorption or nourish*).tw,kw.
42	(diet? or dietary or nutrition* or supplement* or food? or feed*).tw,kw.
43	41 or 42
44	40 and 43
45	limit 44 to yr = “1997 ‐Current”
46	limit 45 to English language

### Web of Science

Science Citation Index Expanded (SCI‐EXPANDED)

Social Sciences Citation Index (SSCI)

Conference Proceedings Citation Index‐Science (CPCI‐S)

Conference Proceedings Citation Index‐ Social Science & Humanities (CPCI‐SSH)

Emerging Sources Citation Index (ESCI)

Search Strategy:

**Table:**

# 43		#40 AND #37
		Refined by: PUBLICATION YEARS: ( 2017 OR 2008 OR 1999 OR 2016 OR 2007 OR 2000 OR 2015 OR 2006 OR 1997 OR 2014 OR 2018 OR 1998 OR 2013 OR 2005 OR 2012 OR 2002 OR 2011 OR 2004 OR 2010 OR 2003 OR 2009 OR 2001) AND LANGUAGES: ( ENGLISH)
		Indexes = SCI‐EXPANDED, SSCI, CPCI‐S, CPCI‐SSH, ESCI Timespan = All years
# 42		#40 AND #37
		Refined by: PUBLICATION YEARS: ( 2017 OR 2008 OR 1999 OR 2016 OR 2007 OR 2000 OR 2015 OR 2006 OR 1997 OR 2014 OR 2018 OR 1998 OR 2013 OR 2005 OR 2012 OR 2002 OR 2011 OR 2004 OR 2010 OR 2003 OR 2009 OR 2001)
		Indexes = SCI‐EXPANDED, SSCI, CPCI‐S, CPCI‐SSH, ESCI Timespan = All years
# 41		#40 AND #37
		Indexes = SCI‐EXPANDED, SSCI, CPCI‐S, CPCI‐SSH, ESCI Timespan = All years
# 40		#39 OR #38
		Indexes = SCI‐EXPANDED, SSCI, CPCI‐S, CPCI‐SSH, ESCI Timespan = All years
# 39		TS = (diet? or dietary or nutrition* or supplement* or food? or feed*)
		Indexes = SCI‐EXPANDED, SSCI, CPCI‐S, CPCI‐SSH, ESCI Timespan = All years
# 38		TS = (“macro‐nutrient” or “macro nutrient” or macronutrient or malnutrition or malnourishment or intracortical* or undernutrition or undernourishment or underfeeding or malabsorption or nourish*)
		Indexes = SCI‐EXPANDED, SSCI, CPCI‐S, CPCI‐SSH, ESCI Timespan = All years
# 37		#36 OR #30 OR #29
		Indexes = SCI‐EXPANDED, SSCI, CPCI‐S, CPCI‐SSH, ESCI Timespan = All years
# 36		#35 AND #13
		Indexes = SCI‐EXPANDED, SSCI, CPCI‐S, CPCI‐SSH, ESCI Timespan = All years
# 35		#34 OR #33 OR #32 OR #31
		Indexes = SCI‐EXPANDED, SSCI, CPCI‐S, CPCI‐SSH, ESCI Timespan = All years
# 34		TS = (“volunt* worker?” or “peer group?” or “woman* group*” or “women* group*” or “child health day” or “integrated management of childhood illness” or IMCI)
		Indexes = SCI‐EXPANDED, SSCI, CPCI‐S, CPCI‐SSH, ESCI Timespan = All years
# 33		TS = ((community or “community based”) NEAR (program* or network* or mobili?er or “out reach” or outreach or aide? or involvement or participation))
		Indexes = SCI‐EXPANDED, SSCI, CPCI‐S, CPCI‐SSH, ESCI Timespan = All years
# 32		TS = ((“health care” or healthcare or health) NEAR (worker? or personnel or volunteer* or provider* or network or campaign* or alliance))
		Indexes = SCI‐EXPANDED, SSCI, CPCI‐S, CPCI‐SSH, ESCI Timespan = All years
# 31		TS = (“community health” NEAR (service? or centre? or center? or promotion or “out reach” or outreach))
		Indexes = SCI‐EXPANDED, SSCI, CPCI‐S, CPCI‐SSH, ESCI Timespan = All years
# 30		#24 AND #13
		Indexes = SCI‐EXPANDED, SSCI, CPCI‐S, CPCI‐SSH, ESCI Timespan = All years
# 29		#28 AND #13
		Indexes = SCI‐EXPANDED, SSCI, CPCI‐S, CPCI‐SSH, ESCI Timespan = All years
# 28		#27 OR #26 OR #25
		Indexes = SCI‐EXPANDED, SSCI, CPCI‐S, CPCI‐SSH, ESCI Timespan = All years
# 27		TS = (“food insecurity*” or “food security” or “food suppl*” or “social protection”)
		Indexes = SCI‐EXPANDED, SSCI, CPCI‐S, CPCI‐SSH, ESCI Timespan = All years
# 26		TS = (“mobile money” or “mobile pay” or “e‐transfer” or “etranfer” or “financial assistance” or “financial support” or “food stamp?” or “food voucher?” or “food assistance” or “cash credit”)
		Indexes = SCI‐EXPANDED, SSCI, CPCI‐S, CPCI‐SSH, ESCI Timespan = All years
# 25		TS = ((cash or money or monetary or electronic) NEAR (transfer* or assistant* or voucher* or allowance?))
		Indexes = SCI‐EXPANDED, SSCI, CPCI‐S, CPCI‐SSH, ESCI Timespan = All years
# 24		#23 OR #22 OR #21 OR #20 OR #19 OR #18 OR #17 OR #16 OR #15 OR #14
		Indexes = SCI‐EXPANDED, SSCI, CPCI‐S, CPCI‐SSH, ESCI Timespan = All years
# 23		TS = (mhealth or “m‐health” or ehealth or “e‐health” or “mobile medicine” or “mobile health”)
		Indexes = SCI‐EXPANDED, SSCI, CPCI‐S, CPCI‐SSH, ESCI Timespan = All years
# 22		TS = (mobile NEAR/2 communication)
		Indexes = SCI‐EXPANDED, SSCI, CPCI‐S, CPCI‐SSH, ESCI Timespan = All years
# 21		TS = ((car or cell or cellular or mobile or smart) NEAR (phone or phones or telephone*))
		Indexes = SCI‐EXPANDED, SSCI, CPCI‐S, CPCI‐SSH, ESCI Timespan = All years
# 20		TS = ((text or short or instant or multimedia or “multi‐media”) NEAR messag*)
		Indexes = SCI‐EXPANDED, SSCI, CPCI‐S, CPCI‐SSH, ESCI Timespan = All years
# 19		TS = (sms or “short messag*” or text* or “mobile outreach” or “mobile out reach” or mms or smartphone* or telephone* or cellphone*)
		Indexes = SCI‐EXPANDED, SSCI, CPCI‐S, CPCI‐SSH, ESCI Timespan = All years
# 18		TS = (“push technolog*” or “web 2.0” or “web 2.0 s” or radio or television* or TV or TVs or twitter or facebook or WeChat or weibo or YouTube or whatsapp or blogging or blog? or “hash tag*” or hashtag* or microblog* or “chat room” or “Internet chat” or “online chat” or email or wiki*)
		Indexes = SCI‐EXPANDED, SSCI, CPCI‐S, CPCI‐SSH, ESCI Timespan = All years
# 17		TS = (phone NEAR/2 (app? or application?))
		Indexes = SCI‐EXPANDED, SSCI, CPCI‐S, CPCI‐SSH, ESCI Timespan = All years
# 16		TS = (“mass media” or “social media” or “social medium?” or “social network*” or sns or multimedia)
		Indexes = SCI‐EXPANDED, SSCI, CPCI‐S, CPCI‐SSH, ESCI Timespan = All years
# 15		TS = ((virtual or online) NEAR/2 (communit* or network* or forum? or support*))
		Indexes = SCI‐EXPANDED, SSCI, CPCI‐S, CPCI‐SSH, ESCI Timespan = All years
# 14		TS = (Internet or “cyber space” or cyberspace or “world wide web” or “information and communication technologies” or “communication technolog*” or ICTs or “communication application*” or “communication apps”)
		Indexes = SCI‐EXPANDED, SSCI, CPCI‐S, CPCI‐SSH, ESCI Timespan = All years
# 13		#12 AND #3
		Indexes = SCI‐EXPANDED, SSCI, CPCI‐S, CPCI‐SSH, ESCI Timespan = All years
# 12		#11 OR #10 OR #9 OR #8 OR #7 OR #6 OR #5 OR #4
		Indexes = SCI‐EXPANDED, SSCI, CPCI‐S, CPCI‐SSH, ESCI Timespan = All years
# 11		TS = (“transitional countr*”)
		Indexes = SCI‐EXPANDED, SSCI, CPCI‐S, CPCI‐SSH, ESCI Timespan = All years
# 10		TS = (lmic or lmics or “third world” or “lami countr*”)
		Indexes = SCI‐EXPANDED, SSCI, CPCI‐S, CPCI‐SSH, ESCI Timespan = All years
# 9		TS = (low NEAR/3 middle NEAR/3 countr*)
		Indexes = SCI‐EXPANDED, SSCI, CPCI‐S, CPCI‐SSH, ESCI Timespan = All years
# 8		TS = (low* NEAR (gdp or gnp or “gross domestic” or “gross national”))
		Indexes = SCI‐EXPANDED, SSCI, CPCI‐S, CPCI‐SSH, ESCI Timespan = All years
# 7		TS = ((developing or “less* developed” or “under developed” or underdeveloped or “middle income” or “low* income”) NEAR (economy or economies))
		Indexes = SCI‐EXPANDED, SSCI, CPCI‐S, CPCI‐SSH, ESCI Timespan = All years
# 6		TS = ((developing or “less* developed” or “under developed” or underdeveloped or “middle income” or “low* income” or underserved or “under served” or deprived or poor*) NEAR (countr* or nation? or population? or world))
		Indexes = SCI‐EXPANDED, SSCI, CPCI‐S, CPCI‐SSH, ESCI Timespan = All years
# 5		TS = (Afghanistan or Albania or Algeria or Angola or Antigua or Barbuda or Argentina or Armenia or Armenian or Aruba or Azerbaijan or Bahrain or Bangladesh or Barbados or Benin or Byelarus or Byelorussian or Belarus or Belorussian or Belorussia or Belize or Bhutan or Bolivia or Bosnia or Herzegovina or Hercegovina or Botswana or Brasil or Brazil or Bulgaria or “Burkina Faso” or “Burkina Fasso” or “Upper Volta” or Burundi or Urundi or Cambodia or “Khmer Republic” or Kampuchea or Cameroon or Cameroons or Cameron or Camerons or “Cape Verde” or “Central African Republic” or Chad or Chile or China or Colombia or Comoros or “Comoro Islands” or Comores or Mayotte or Congo or Zaire or “Costa Rica” or “Cote d'Ivoire” or “Ivory Coast” or Croatia or Cuba or Cyprus or Czechoslovakia or “Czech Republic” or Slovakia or “Slovak Republic” or Djibouti or “French Somaliland” or Dominica or “Dominican Republic” or “East Timor” or “East Timur” or “Timor Leste” or Ecuador or Egypt or “United Arab Republic” or “El Salvador” or Eritrea or Estonia or Ethiopia or Fiji or Gabon or “Gabonese Republic” or Gambia or Gaza or “Georgia Republic” or “Georgian Republic” or Ghana or “Gold Coast” or Greece or Grenada or Guatemala or Guinea or Guam or Guiana or Guyana or Haiti or Honduras or Hungary or India or Maldives or Indonesia or Iran or Iraq or “Isle of Man” or Jamaica or Jordan or Kazakhstan or Kazakh or Kenya or Kiribati or Korea or Kosovo or Kyrgyzstan or Kirghizia or “Kyrgyz Republic” or Kirghiz or Kirgizstan or “Lao PDR” or Laos or Latvia or Lebanon or Lesotho or Basutoland or Liberia or Libya or Lithuania or Macedonia or Madagascar or “Malagasy Republic” or Malaysia or Malaya or Malay or Sabah or Sarawak or Malawi or Nyasaland or Mali or Malta or “Marshall Islands” or Mauritania or Mauritius or “Agalega Islands” or Mexico or Micronesia or “Middle East” or Moldova or Moldovia or Moldovian or Mongolia or Montenegro or Morocco or Ifni or Mozambique or Myanmar or Myanma or Burma or Namibia or Nepal or “Netherlands Antilles” or “New Caledonia” or Nicaragua or Niger or Nigeria or “Northern Mariana Islands” or Oman or Muscat or Pakistan or Palau or Palestine or Panama or Paraguay or Peru or Philippines or Philipines or Phillipines or Phillippines or Poland or Portugal or Puerto Rico or Romania or Rumania or Roumania or Russia or Russian or Rwanda or Ruanda or “Saint Kitts” or “St Kitts” or Nevis or “Saint Lucia” or “St Lucia” or “Saint Vincent” or “St Vincent” or Grenadines or Samoa or “Samoan Islands” or “Navigator Island” or “Navigator Islands” or “Sao Tome” or “Saudi Arabia” or Senegal or Serbia or Montenegro or Seychelles or “Sierra Leone” or Slovenia or “Sri Lanka” or Ceylon or “Solomon Islands” or Somalia or “South Africa” or Sudan or Suriname or Surinam or Swaziland or Syria or Tajikistan or Tadzhikistan or Tadjikistan or Tadzhik or Tanzania or Thailand or Togo or “Togolese Republic” or Tonga or Trinidad or Tobago or Tunisia or Turkey or Turkmenistan or Turkmen or Uganda or Ukraine or Uruguay or USSR or “Soviet Union” or “Union of Soviet Socialist Republics” or Uzbekistan or Uzbek or Vanuatu or “New Hebrides” or Venezuela or Vietnam or “Viet Nam” or “West Bank” or Yemen or Yugoslavia or Zambia or Zimbabwe or Rhodesia)
		Indexes = SCI‐EXPANDED, SSCI, CPCI‐S, CPCI‐SSH, ESCI Timespan = All years
# 4		TS = (Africa or Asia or Caribbean or “West Indies” or “South America” or “Latin America” or “Central America”)
		Indexes = SCI‐EXPANDED, SSCI, CPCI‐S, CPCI‐SSH, ESCI Timespan = All years
# 3		#2 OR #1
		Indexes = SCI‐EXPANDED, SSCI, CPCI‐S, CPCI‐SSH, ESCI Timespan = All years
# 2		TS = (obstetric* or gynecolog* or gynaecolog* or perinatal or prenatal or “pre natal” or antenatal or “ante natal” or postnatal or “post natal” or “maternal health” or gestation or pregnancies or pregnant or pregnancy or childbearing or gravidity or mother* or breastfeed* or “breast feeding” or woman or women)
		Indexes = SCI‐EXPANDED, SSCI, CPCI‐S, CPCI‐SSH, ESCI Timespan = All years
# 1		TS = (pediatric* or paediatric* or child* or newborn* or congenital* or infan* or baby or babies or neonat* or “pre‐term” or preterm* or “premature birth*” or NICU or preschool* or “pre‐school*” or kindergarten* or kindergarden* or “elementary school*” or “nursery school*” or (“day care*” NOT adult*) or schoolchild* or toddler* or boy or boys or girl* or “middle school*” or pubescen* or juvenile* or teen* or youth* or “high school*” or adolesc* or “pre‐pubesc*” or prepubesc*)
		Indexes = SCI‐EXPANDED, SSCI, CPCI‐S, CPCI‐SSH, ESCI Timespan = All years

**EBM Reviews ‐ Cochrane Central Register of Controlled Trials**

Search Strategy:

**Table:**

#	Searches
1	(pediatric* or paediatric* or child* or newborn* or congenital* or infan* or baby or babies or neonat* or “pre‐term” or preterm* or “premature birth*” or NICU or preschool* or “pre‐school*” or kindergarten* or kindergarden* or “elementary school*” or “nursery school*” or (“day care*” not adult*) or schoolchild* or toddler* or boy or boys or girl* or “middle school*” or pubescen* or juvenile* or teen* or youth* or “high school*” or adolesc* or “pre‐pubesc*” or prepubesc*).tw,kf. or (child* or adolesc* or pediat* or paediat*).jn.
2	Prenatal care/ or Perinatal care/ or obstetrics/ or breast feeding/
3	women/ or pregnant women/
4	(obstetric* or gynecolog* or gynaecolog* or perinatal or prenatal or “pre natal” or antenatal or “ante natal” or postnatal or “post natal” or “maternal health” or gestation or pregnancies or pregnant or pregnancy or childbearing or gravidity or mother* or breastfeed* or “breast feeding” or woman or women).tw,kf.
5	or/1–4
6	Developing Countries.sh,kf.
7	(Africa or Asia or Caribbean or “West Indies” or “South America” or “Latin America” or “Central America”).tw,kf,hw,cp.
8	(Afghanistan or Albania or Algeria or Angola or Antigua or Barbuda or Argentina or Armenia or Armenian or Aruba or Azerbaijan or Bahrain or Bangladesh or Barbados or Benin or Byelarus or Byelorussian or Belarus or Belorussian or Belorussia or Belize or Bhutan or Bolivia or Bosnia or Herzegovina or Hercegovina or Botswana or Brasil or Brazil or Bulgaria or “Burkina Faso” or “Burkina Fasso” or “Upper Volta” or Burundi or Urundi or Cambodia or “Khmer Republic” or Kampuchea or Cameroon or Cameroons or Cameron or Camerons or “Cape Verde” or “Central African Republic” or Chad or Chile or China or Colombia or Comoros or “Comoro Islands” or Comores or Mayotte or Congo or Zaire or “Costa Rica” or “Cote d'Ivoire” or “Ivory Coast” or Croatia or Cuba or Cyprus or Czechoslovakia or “Czech Republic” or Slovakia or “Slovak Republic” or Djibouti or “French Somaliland” or Dominica or “Dominican Republic” or “East Timor” or “East Timur” or “Timor Leste” or Ecuador or Egypt or “United Arab Republic” or “El Salvador” or Eritrea or Estonia or Ethiopia or Fiji or Gabon or “Gabonese Republic” or Gambia or Gaza or “Georgia Republic” or “Georgian Republic” or Ghana or “Gold Coast” or Greece or Grenada or Guatemala or Guinea or Guam or Guiana or Guyana or Haiti or Honduras or Hungary or India or Maldives or Indonesia or Iran or Iraq or “Isle of Man” or Jamaica or Jordan or Kazakhstan or Kazakh or Kenya or Kiribati or Korea or Kosovo or Kyrgyzstan or Kirghizia or “Kyrgyz Republic” or Kirghiz or Kirgizstan or “Lao PDR” or Laos or Latvia or Lebanon or Lesotho or Basutoland or Liberia or Libya or Lithuania or Macedonia or Madagascar or “Malagasy Republic” or Malaysia or Malaya or Malay or Sabah or Sarawak or Malawi or Nyasaland or Mali or Malta or “Marshall Islands” or Mauritania or Mauritius or “Agalega Islands” or Mexico or Micronesia or “Middle East” or Moldova or Moldovia or Moldovian or Mongolia or Montenegro or Morocco or Ifni or Mozambique or Myanmar or Myanma or Burma or Namibia or Nepal or “Netherlands Antilles” or “New Caledonia” or Nicaragua or Niger or Nigeria or “Northern Mariana Islands” or Oman or Muscat or Pakistan or Palau or Palestine or Panama or Paraguay or Peru or Philippines or Philipines or Phillipines or Phillippines or Poland or Portugal or Puerto Rico or Romania or Rumania or Roumania or Russia or Russian or Rwanda or Ruanda or “Saint Kitts” or “St Kitts” or Nevis or “Saint Lucia” or “St Lucia” or “Saint Vincent” or “St Vincent” or Grenadines or Samoa or “Samoan Islands” or “Navigator Island” or “Navigator Islands” or “Sao Tome” or “Saudi Arabia” or Senegal or Serbia or Montenegro or Seychelles or “Sierra Leone” or Slovenia or “Sri Lanka” or Ceylon or “Solomon Islands” or Somalia or “South Africa” or Sudan or Suriname or Surinam or Swaziland or Syria or Tajikistan or Tadzhikistan or Tadjikistan or Tadzhik or Tanzania or Thailand or Togo or “Togolese Republic” or Tonga or Trinidad or Tobago or Tunisia or Turkey or Turkmenistan or Turkmen or Uganda or Ukraine or Uruguay or USSR or “Soviet Union” or “Union of Soviet Socialist Republics” or Uzbekistan or Uzbek or Vanuatu or “New Hebrides” or Venezuela or Vietnam or “Viet Nam” or “West Bank” or Yemen or Yugoslavia or Zambia or Zimbabwe or Rhodesia).hw,kf,it,ab,cp.
9	((developing or “less* developed” or “under developed” or underdeveloped or “middle income” or “low* income” or underserved or “under served” or deprived or poor*) adj1 (countr* or nation? or population? or world)).tw.
10	((developing or “less* developed” or “under developed” or underdeveloped or “middle income” or “low* income”) adj1 (economy or economies)).tw.
11	(low* adj1 (gdp or gnp or “gross domestic” or “gross national”)).tw.
12	(low adj3 middle adj3 countr*).tw.
13	(lmic or lmics or “third world” or “lami countr*”).tw.
14	“transitional countr*”.tw.
15	or/6–14
16	5 and 15
17	exp mass media/ or exp social media/ or blogging/ or social networking/ or radio/
18	(Internet or “cyber space” or cyberspace or “world wide web” or “information and communication technologies” or “communication technolog*” or ICTs or “communication application*” or “communication apps”).tw,kf.
19	((virtual or online) adj2 (communit* or network* or forum? or support*)).tw,kf.
20	(“mass media” or “social media” or “social medium?” or “social network*” or sns or multimedia).tw,kf.
21	(phone adj2 (app? or application?)).tw,kf.
22	(“push technolog*” or “web 2.0” or “web 2.0 s” or radio or television* or TV or TVs or twitter or facebook or WeChat or weibo or YouTube or whatsapp or blogging or blog? or “hash tag*” or hashtag* or microblog* or “chat room” or “Internet chat” or “online chat” or email or wiki*).tw,kf.
23	(sms or “short messag*” or text* or “mobile outreach” or “mobile out reach” or mms or smartphone* or telephone* or cellphone*).tw,kf.
24	((text or short or instant or multimedia or “multi‐media”) adj1 messag*).tw,kf.
25	((car or cell or cellular or mobile or smart) adj1 (phone or phones or telephone*)).tw,kf.
26	(mobile adj2 communication).tw,kf.
27	(mhealth or “m‐health” or ehealth or “e‐health” or “mobile medicine” or “mobile health”).tw,kf.
28	or/17–27
29	16 and 28
30	((cash or money or monetary or electronic) adj1 (transfer* or assistant* or voucher* or allowance?)).tw,kf.
31	(“mobile money” or “mobile pay” or “e‐transfer” or “etranfer” or “financial assistance” or “financial support” or “food stamp?” or “food voucher?” or “food assistance” or “cash credit”).tw,kf.
32	food supply/ or (“food insecurity*” or “food security” or “food suppl*” or “social protection”).tw,kf.
33	or/30–32
34	16 and 33
35	(“community health” adj1 (service? or centre? or center? or promotion or “out reach” or outreach)).tw,kf.
36	((“health care” or healthcare or health) adj1 (worker? or personnel or volunteer* or provider* or network or campaign* or alliance)).tw,kf.
37	((community or “community based”) adj1 (program* or network* or mobili?er or “out reach” or outreach or aide? or involvement or participation)).tw,kf.
38	(“volunt* worker?” or “peer group?” or “wom#n group?” or “wom#n's group?” or “child health day” or “integrated management of childhood illness” or IMCI).tw,kf.
39	or/35–38
40	1089
41	29 or 34 or 40
42	(“macro‐nutrient” or “macro nutrient” or macronutrient or malnutrition or malnourishment or intracortical* or undernutrition or undernourishment or underfeeding or malabsorption or nourish*).tw,kf.
43	(diet? or dietary or nutrition* or supplement* or food? or feed*).tw,kf.
44	or/42–43
45	41 and 44
46	limit 45 to yr = “1997 ‐Current”
47	limit 46 to english language
